# Enhancing Acclimatization Conditions for *Vriesea splendens* ‘Fire’: A Comparative Analysis of Substrate Effects on Growth and Survival

**DOI:** 10.3390/plants14020172

**Published:** 2025-01-09

**Authors:** Eman Abdelhakim Eisa, Daniela Salome Pasquel Davila, Máté Ördögh

**Affiliations:** 1 Department of Floriculture and Dendrology, Institute of Landscape Architecture, Urban Planning and Garden Art, Hungarian University of Agriculture and Life Sciences (MATE), Villányi Street 29-43, 1118 Budapest, Hungary; abdelhakim.eman1@gmail.com; 2 Botanical Gardens Research Department, Horticulture Research Institute, Agricultural Research Center (ARC), Giza 12619, Egypt; 3 Yachay Botanical Garden, Yachay Tech University, Hacienda San Eloy, Urcuquí 100115, Ecuador; daniela.pasquel.d@gmail.com

**Keywords:** bromeliad propagation, *Vriesea splendens*, micropropagation, acclimatization, substrates, coco coir efficiency

## Abstract

This study investigates the acclimatization success of *Vriesea splendens* ’Fire’, a popular ornamental bromeliad, through in vitro propagation on various substrates. Due to the increasing demand for *V. splendens*, micropropagation offers a promising solution to overcome the limitations of traditional propagation methods. In this research, acclimatization was conducted in two trial types: in the one-step greenhouse conditions, and in two-step acclimatization, which introduced a controlled laboratory step before transferring plants to the greenhouse. The substrates examined included pure and mixed forms of turf, perlite, coco coir, pine bark (hereafter referred to as bark), moss, and vermiculite. Morphological traits such as plant height, leaf length, number and length of roots, and fresh weight were evaluated, together with physiological parameters, such as chlorophyll and carotenoid concentrations and survival percentage, to test the effectiveness of acclimatization. Coco coir-based substrates significantly enhanced plant height, root development, and survival percentages in both experiments compared with other substrates, thus proving its suitability for the propagation of *V. splendens*. Vermiculite had the highest survival rate during one-step acclimatization, whereas turf showed a very good performance in two-step acclimatization. On the opposite side, substrates containing bark and moss showed a reduced effect on plant growth and survival, which indicated the vital role of substrates for best development. Statistical analyses confirmed the superiority of some combinations of substrates related to physiological health, showing that optimal acclimatization results could be improved by a chosen substrate. These results strengthen the present in vitro propagation protocols of the *Vriesea* species by confirming the relevance of substrate choice in producing hardy plants with good commercial prospects.

## 1. Introduction

*Vriesea splendens* ‘Fire’, an eye-catching plant in the *Bromeliaceae* family, originates from the tropical rainforests of South America, particularly in regions such as Trinidad, French Guiana, Guyana, Suriname, and Venezuela. Initially described in 1850, it is also recognized by its synonym, *Tillandsia splendens* Brongn [1]. As an angiosperm, *V. splendens* belongs to the diverse subfamily *Tillandsioideae*, which includes approximately 1220 species across nine genera [2,3]. Its taxonomy is Tropicos, 2021, and it belongs to the kingdom *Plantae*, the family *Bromeliaceae*, the subfamily *Tillandsioideae*, the genus *Vriesea*, and the species *Vriesea splendens*.

The species stands out morphologically, with several unique adaptations. The leaves of the plant are arranged in a rosette structure, forming a kind of natural reservoir [4], enabling the organism to store water and absorb nutrients from decaying organic material [5]. The latter is increased by the presence of special trichomes on the undersides of the leaves, while its xeromorphic features are adaptations to ancestral dry environments [6]. The leaves of *V. splendens* are glabrous with striking coloration, displaying green and dark-green horizontal stripes [7]. Moreover, the species has striking showy inflorescences, with brilliant red bracts and tubular flowers ranging in color from yellow to white and green. These attractive features have made it a popular ornamental plant in Europe, which requires efficient propagation methods to supply the market demand [7].

Bromeliads are appreciated worldwide within the floriculture industry; species of *Vriesea* are very popular as ornamental plants. Their hybridization, including with *Tillandsia* and *Guzmania*, has been carried out to bring new varieties into the market [8]. However, their wide ornamental use and illicit collection threaten their wild populations, especially in Latin America [9]. As a consequence of habitat degradation and illegal collection, *Vriesea splendens* has been considered vulnerable in several Latin American countries, such as Venezuela and Brazil [10,11]. Conservation strategies include in situ habitat preservation and ex situ seed and tissue banking, contributing to safeguarding long-term genetic conservation [12]. Cultivation in controlled conditions also contributes to its conservation by reducing pressure on wild populations.

Three propagation methods exist for *V. splendens*—sexual, asexual, and micropropagation—which all have different characteristics and requirements. Environmental conditions such as light, temperature, humidity, and substrate regulate sexual propagation in bromeliads, and therefore in *V. splendens* [13]. Consequently, pollination—a strict requirement for this process—can be effected by many pollinator species that include hummingbirds, bats, bees and butterflies, as its seeds are dispersed by the wind. Larger inflorescences may increase potential seed production [14]. *Vriesea* plants also reproduce asexually by producing clonal growth at the axillary buds. The plant’s method supports its site retention and increases its local dominance, replacing the mother plant as it dies after seed dispersal [15,16]. Despite the lack of extensive investigation into *Vriesea splendens*, micropropagation is a very effective method for the mass propagation and ex situ conservation of threatened species of bromeliads. The process generally involves germinating seeds in nutrient-enriching media and transferring the young shoots to new media after several weeks. Critical parameters such as chlorophyll content, peroxidase activity, shoot number, plant height, fresh weight, root number, and acclimatization survival rates are used to monitor success in propagation [17,18].

Acclimatization studies for the *Vriesea* genus represent a critical final step in micropropagation research, aimed at enhancing the production of species with significant ornamental value, particularly those threatened by illegal harvesting. Significant focus has been given to *Vriesea reitzii*, native to the Brazilian Atlantic Forest, where the improvement of in vitro micropropagation techniques is essential. Studies have tested a variety of hormonal applications, showing that gibberellic acid (GA3) results in higher survival rates in the acclimatization stage compared to other hormones, like α-naphthaleneacetic acid (NAA) and 6-benzylaminopurine (BAP) [19,20]. Despite the acclimatization stage being such a critical stage, there is a surprising lack of comparative studies on substrate conditions, especially regarding pH level. For example, in *Vriesea philippocoburgii*, substrate pH manipulation using sulfur showed that, although survival was high, some treatments severely damaged the plants [21]. Also, environmental factors such as temperature [22], sucrose levels [23,24], and hormone applications [18] significantly influence acclimatization success by promoting root formation, plant vigor, and photosynthetic activity. Additionally, the modification of misting, humidity, and light conditions plays a more significant role in helping plantlets adjust to ambient environments by gradually modifying their physiological and anatomical traits. For example, the use of mist reactors and controlled humidity environments has been shown to improve the survival and quality of plantlets, by reducing hyperhydration and promoting better stomatal regulation [25,26]. Similarly, supplementary lighting has been found to enhance leaf thickness and photosynthesis rates, which are vital for acclimation [27]. Moreover, the choice of potting substrate is crucial for plantlet acclimation, due to its role in supporting root development while providing essential nutrients and stability [28,29]. The potting substrate acts as the foundation for the plant’s growth, influencing water retention, aeration, and nutrient availability, which are essential for the successful transition from in vitro to ex vitro conditions. For instance, a study on *Telopea speciosissima* highlighted that a peat and perlite mixture was superior for rooting microshoots due to its air-filled porosity, which is crucial for root development and acclimatization [30].

In summary, the results highlight the necessity for customized acclimatization approaches to enhance the growth and survival rates of *Vriesea species,* in both their natural habitats and cultivated settings.

This study builds upon previous research regarding acclimatization methods, which focuses on the evaluation of the acclimatization success of *V. splendens* ’Fire’ on various substrate variations. Specifically, it aims to consider the influence of different substrates on morphological traits, as well as the levels of chlorophyll and carotenoid pigments, in addition to general survival rates. By identifying the optimal substrate conditions under controlled greenhouse conditions as a one-step acclimation and under laboratory conditions as part of a two-step acclimation, the present in-depth review aims to identify the optimal substrate conditions for the acclimatization and establishment of *V. splendens* ’Fire’ under controlled environments.

## 2. Results

A multivariate analysis of variance (MANOVA) was conducted to evaluate the effect of substrate type on vegetative growth parameters and chlorophyll and carotenoid contents during the first and second acclimatization experiments. The analysis revealed the significant effects of substrate type on the dependent variables.

For the first acclimatization experiment, significant effects were observed for vegetative growth parameters at both the beginning (Wilks’ Lambda = 0.415, F(140, 4010.065) = 4.050, *p* < 0.001) and the end of the experiment (Wilks’ Lambda = 0.276, F(140, 3325.792) = 5.085, *p* < 0.001). Similarly, significant effects were found for pigment contents at the beginning (Wilks’ Lambda = 0.037, F(40, 82) = 8.637, *p* < 0.001) and the end (Wilks’ Lambda = 0.069, F(40, 82) = 5.742, *p* < 0.001).

In the second acclimatization experiment, the substrate type significantly influenced vegetative growth parameters at the beginning (Wilks’ Lambda = 0.475, F(98, 2722.155) = 3.469, *p* < 0.001) and at the end of the experiment (Wilks’ Lambda = 0.129, F(98, 2083.375) = 8.135, *p* < 0.001). Significant effects were also observed for pigment contents at the beginning (Wilks’ Lambda = 0.191, F(26, 60) = 2.972, *p* < 0.001) and the end (Wilks’ Lambda = 0.098, F(26, 60) = 5.072, *p* < 0.001).

Post hoc analyses provided detailed pairwise comparisons, revealing significant differences among substrate treatments. These findings underscore the substantial impact of substrate type on the dynamics of the dependent variables across both acclimatization experiments and time points.

### 2.1. Morphological and Physiological Characteristics

#### 2.1.1. Number of Shoots

##### First Trial (One-Step Acclimatization in Greenhouse Conditions)

**Figure 1:**
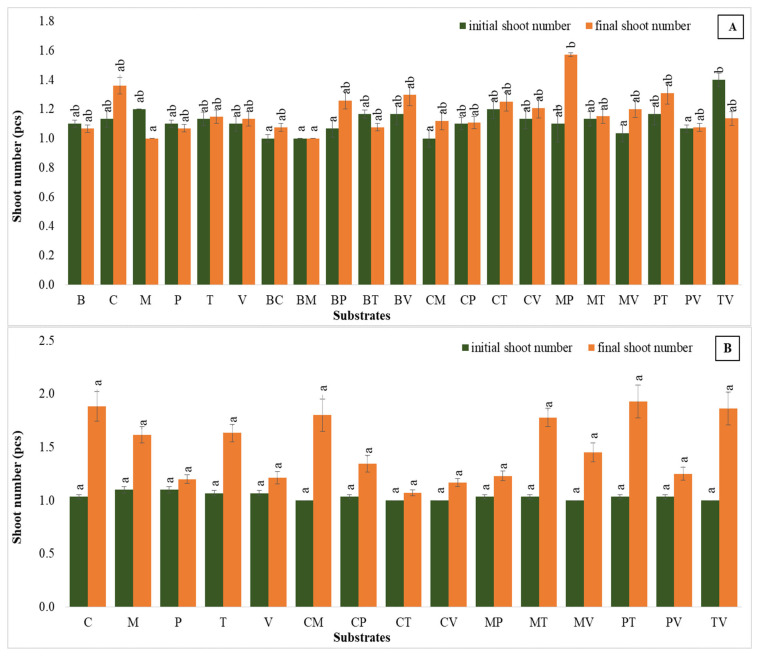
The shoot numbers in the first (**A**) and second (**B**) acclimatization trials, with the use of different substrates. In all graphs, distinct small vertical letters denote significant differences across treatments (Games–Howell, *p* < 0.05). Abbreviations with large, horizontal letters: (B) bark, (C) coco coir, (M) moss, (P) perlite, (T) turf, and (V) vermiculite. Groups with two letters show the combinations of these substrates.

Shoot numbers varied across different substrates in greenhouse acclimatization (Figure 1A). While statistical analysis did not indicate significant differences among treatments, some substrates showed a positive trend in shoot number increases. For instance, the combination of moss + perlite demonstrated the most substantial improvement, with shoot numbers increasing from 1.10 to 1.57, representing a 43% increase. This was followed by coco coir, which showed a notable rise, from 1.13 to 1.36 (20% improvement). The mixture of bark + perlite also displayed an increase, from 1.07 to 1.26 (17.8% improvement). Conversely, some substrates, such as moss, perlite, bark + turf, bark + moss and turf + vermiculite, exhibited no growth or decline.

#### 2.1.2. Second Trial (Two–Step Acclimatization)

This was performed under controlled laboratory conditions before transferring to the greenhouse, and the turf-based mixtures, such as perlite + turf and turf + vermiculite, emerged as the most effective substrates, in which shoot numbers nearly doubled (1.87-fold increase) from 1.03 to 1.93, and 1.00 to 1.86. Coco coir showed the second-highest growth with an 83% increase, rising from 1.03 to 1.88, followed by coco coir + moss and moss + turf, which exhibited increases of 80% and 72%, respectively (Figure 1B).

### 2.2. The Plant Height

**Figure 2:**
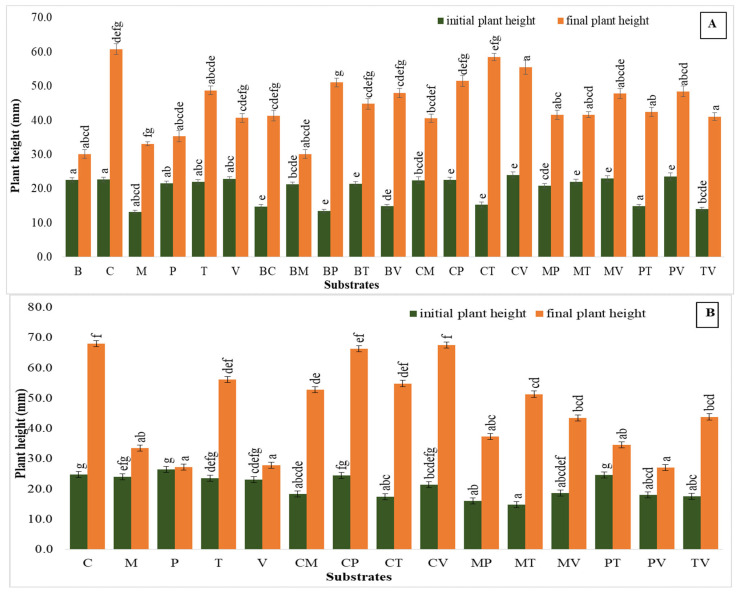
Plant height in the first (**A**) and second (**B**) acclimatization trials, with the use of different substrates. Distinct small vertical letters denote significant differences across treatments (Games–Howell, *p* < 0.05).

In the first trial (Figure 2A), coco coir and its combinations stood out, with coco coir alone showing a 168% increase, and coco coir + vermiculite rising from 24.00 mm to 55.55 mm (132%), while the highest increase in the final plant height compared to the initial height was recorded in bark + perlite and bark + vermiculite, by 278% and 224%, respectively. The combination of coco coir + perlite also performed well, growing from 22.50 mm to 51.46 mm (129%). Conversely, substrates such as perlite alone exhibited a relative improvement in final plant height development, increasing by 64% (21.50 mm to 35.34 mm), followed by bark + moss, which only increased by 41%.

In the second study (Figure 2B), the coco coir-based mixture maintained its dominance, with coco coir + vermiculite achieving the highest growth increase, by 216%, at the final plant height, and coco coir alone following, with an increase from 24.77 cm to 67.96 mm (174%). Turf-based mixtures also showed strong results, such as coco coir + turf, which rose from 17.43 cm to 54.89 mm (215%), and pure turf, which increased from 23.63 mm to 56.20 mm (138%). Moss-based combinations, such as moss + turf (from 14.80 mm to 51.37 mm, 247%) and moss + vermiculite (from 18.63 mm to 43.55 mm, 134%), showed considerable improvements, but moss alone performed less effectively, increasing by only 40%.

**Figure 3:**
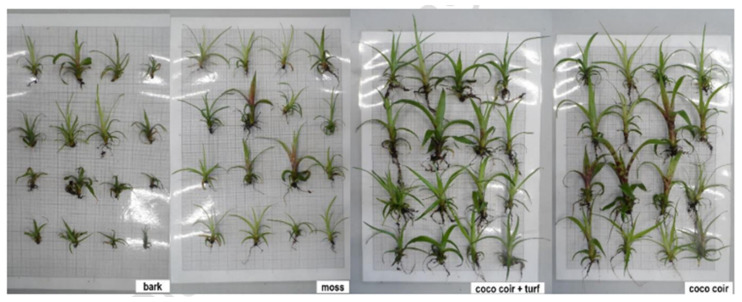
The final plant height in the first acclimatization.

Figure 3 demonstrates visible plant height difference between certain substrates, such as coco coir, bark, moss, and a coco coir + turf mixture.

### 2.3. Plant Fresh Weight

#### 2.3.1. First Trial

**Figure 4:**
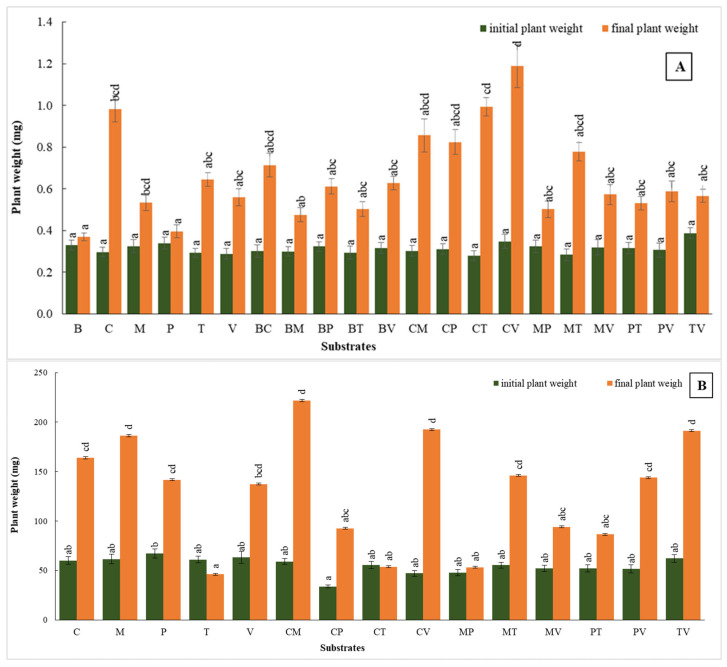
Plant fresh weight in the first (**A**) and second (**B**) acclimatization trials, with the use of different substrates. Distinct small vertical letters denote significant differences across treatments (Games–Howell, *p* < 0.05).

**Figure 5:**
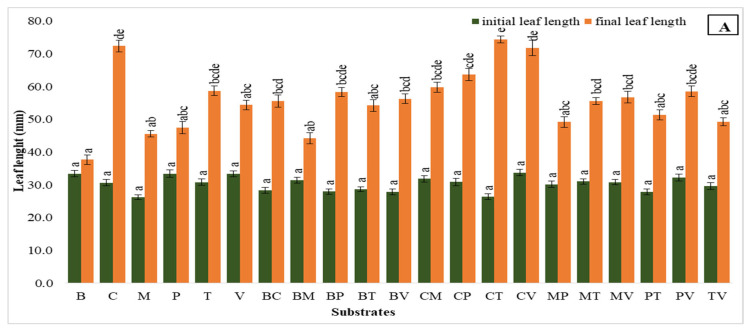

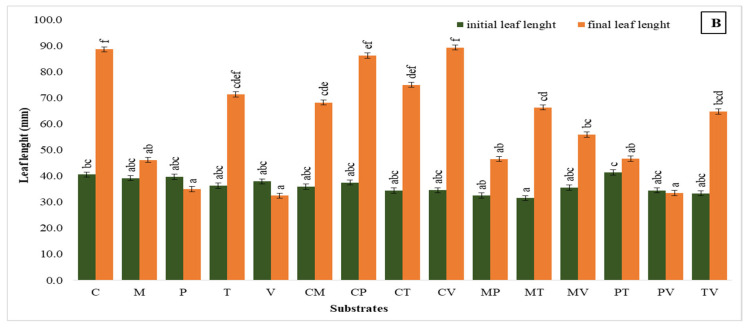
Leaf length in the first (**A**) and second (**B**) acclimatization trials, with the use of different substrates. Distinct small vertical letters denote significant differences across treatments (Games–Howell, *p* < 0.05).

At the beginning phase of this acclimatization trial, the initial plant fresh weight was similar across all substrates. However, notable variability was observed in plant weight gain by the end of the phase, indicating the distinct effects of different substrate compositions on early plant growth. Among the substrates tested, coco coir showed a significant increase in weight, rising from 0.2967 mg to 0.9828 mg, indicating 3.31-fold growth. The coco coir + vermiculite combination displayed exceptional growth, with the initial weight of 0.3480 mg increasing to 1.1879 mg, representing the highest final weight achieved and a 3.42-fold increase (Figure 4A). Other effective combinations included coco coir + perlite and coco coir + turf, both of which showed substantial increases in plant weight, though these were less pronounced than the growth observed in coco coir alone or coco coir + vermiculite (Figure 5). In contrast, bark, perlite, and the perlite mixture exhibited limited growth, with their final weights only being marginally higher than their initial values. This suggests that these substrates may be less effective in supporting the initial acclimatization of *V. splendens*, possibly indicating a reduced ability to promote early plant development under the given conditions.

#### 2.3.2. Second Trial

Substrates continued to significantly influence plant growth. Coco coir + moss exhibited the highest increase, with plant weights rising from 58.97 mg to 221.85 mg, reflecting a 3.76-fold increase and demonstrating a strong synergistic effect that greatly supports plant development (Figure 4B). Pure coco coir also showed substantial growth, with weights increasing from 60.00 mg to 163.97 g, representing a 2.73-fold enhancement. Similarly, moss alone led to a rise from 61.57 mg to 186.30 mg, showing a 3.02-fold increase, highlighting its effectiveness in promoting plant growth. In contrast, the turf and perlite mixtures exhibited limited progress, with turf showing a decrease in weight from 60.93 mg to 46.08 mg, indicating a 24.5% reduction (Figure 4B). This phenomenon suggests that these substrates may be less effective in supporting plant growth during the acclimatization phase under laboratory conditions, potentially limiting their utility in the initial stages of plant development.

### 2.4. Leaf Length

Based on the data provided for the leaf length measurements of *V. splendens* across different substrates during the acclimatization phases, several key observations emerged.

In the first trial, significant growth in leaf length was observed with substrates containing coco coir, coco coir + vermiculite, and coco coir + turf, which showed final leaf lengths of 72.40 mm, 71.86 mm, and 74.46 mm, respectively. These results indicate the effectiveness of coco coir-based substrates in promoting leaf extension during the early stages. Comparatively, bark, perlite, bark + moss, and turf + vermiculite showed less pronounced growth, suggesting that these substrates might not promote leaf elongation as effectively as coco coir + turf or coco coir alone (Figure 5A).

In the second study, coco coir alone and the combination of coco coir + vermiculite continued to outperform the others, with remarkable final leaf lengths of 88.58 mm and 89.27 mm, consequently. Notably, coco coir + perlite also yielded high growth (86.17 mm), affirming coco coir’s role as a key component for enhancing leaf development. In contrast, pure substrates, like vermiculite, perlite, and their mixture, showed a reduction in leaf length over time, with final values of 32.43 mm, 34.95 mm, and 33.42 mm, indicating a potential limitation in their ability to maintain growth in controlled conditions (Figure 5B).

### 2.5. The Number of Roots

The data analysis of root numbers across different substrates during the two acclimatization phases for *V. splendens* reveals key insights into substrate effectiveness.

**Figure 6:**
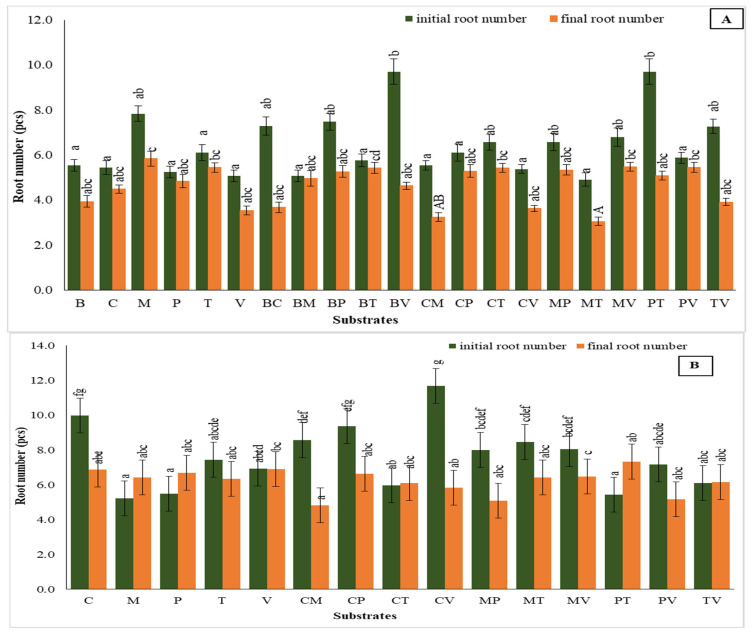
Number of roots in the first (**A**) and second (**B**) acclimatization trials, with the use of different substrates. Distinct small vertical letters denote significant differences across treatments (Games–Howell, *p* < 0.05).

In the first trial, initial root numbers varied substantially among substrates (Figure 6A). Specifically, bark + vermiculite and perlite + turf resulted the highest initial root counts, both at 9.70, yet both showed a significant decrease by the end of the phase, with final root values decreasing by 52% and 47.6%. This decline indicates that, while these substrates may initially support root development, they might not sustain it over time. Moss, with an initial root count of 7.83, also experienced a decrease of 25.5%, ending with a relatively high final root number of 5.83, indicating moderate effectiveness in promoting root retention. Substrates such as coco coir + turf and moss + vermiculite, starting at 6.57 and 6.79, showed more stable root values. Both experienced minor decreases of 17.5% (to 5.42) and 19.3% (to 5.48), highlighting their better capacity to maintain root development throughout the acclimatization.

In the second trial, certain substrates exhibited more consistent root development (Figure 6B). Perlite + turf demonstrated the most significant improvement, with root numbers increasing by approximately 35%, from an initial 5.43 to a final 7.33, highlighting its strong support for root growth in controlled environments. In contrast, coco coir + vermiculite, despite having the highest initial root count of 11.67, experienced a substantial decline of nearly 50%, ending at 5.83. This trend mirrors observations in the first acclimatization type with bark mixtures, which also started with high root counts but failed to sustain growth. Meanwhile, substrates like coco coir, coco coir + perlite, and moss + turf showed either balanced root number increases or minimal decreases, maintaining final values above 6.0. These results suggest their effectiveness in supporting steady root development, making them suitable candidates for the critical transition phase before plants are moved to greenhouse conditions.

### 2.6. Longest Root Lengths

**Figure 7:**
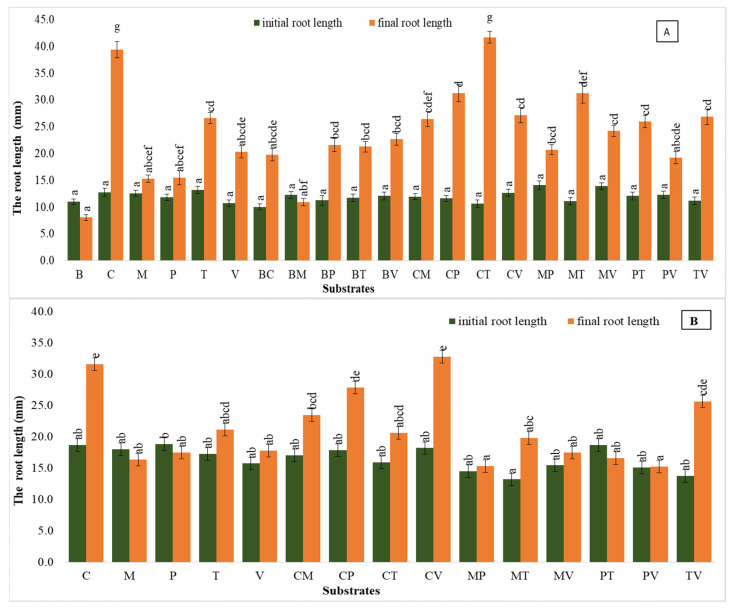
The length of the longest root in the first (**A**) and second (**B**) acclimatization trials, with the use of different substrates. Distinct small vertical letters denote significant differences across treatments (Games–Howell, *p* < 0.05).

In the first study, coco coir + turf demonstrated the most substantial root elongation, with final lengths reaching 41.71 mm, compared to an initial 10.47 mm. Similarly, coco coir recorded the highest final root length at 39.4 mm, displaying the synergistic benefits of these substrates for root extension. Other coco coir mixtures, such as coco coir + perlite and coco coir + vermiculite, also performed well, with final lengths exceeding 31.29 mm and 27.17 mm. In contrast, bark-based substrates, including pure bark (8.07 mm) and bark + moss (10.91 mm), showed the least growth, indicating their limited suitability for root development (Figure 7A).

In the second trial, all coco coir-based substrates supported considerable root growth (particularly coco coir + vermiculite), and coco coir resulted in the longest final root lengths, reaching 32.80 mm and 31.62 mm. Also, turf-based substrates, such as turf (21.13 mm) and turf + vermiculite (25.66 mm), promoted significant root values, underscoring their effectiveness in controlled environments. Conversely, moss and perlite + vermiculite exhibited minimal root elongation, with final lengths below 16 mm, suggesting their limitations in promoting robust root development during this acclimatization study (Figure 7B).

### 2.7. Total Chlorophyll Content

**Figure 8:**
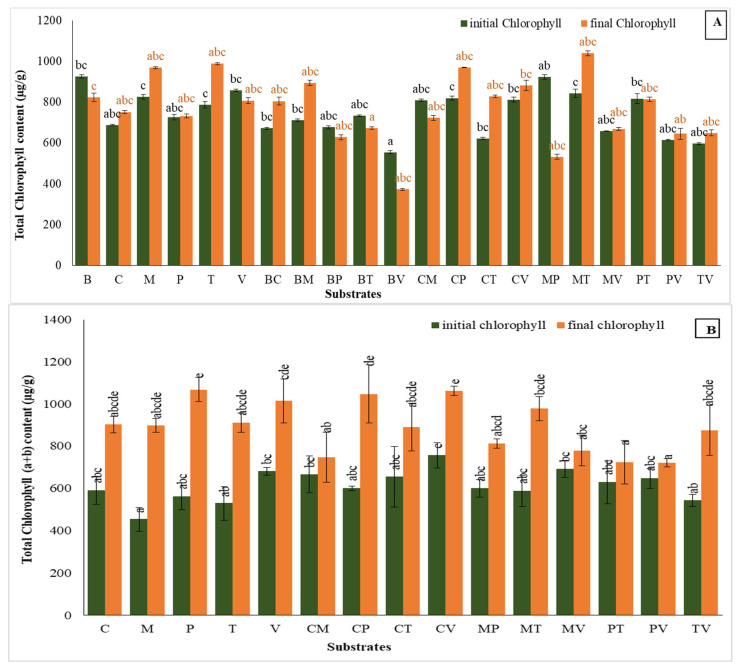
Total chlorophyll content in the first (**A**) and second (**B**) acclimatization trials, with the use of different substrates. Distinct small vertical letters denote significant differences across treatments (Games–Howell, *p* < 0.05).

In the first trial, (Figure 8A), the substrates exhibited varying impacts on the chlorophyll content. Moss showed a noticeable trend in chlorophyll content increase, although this change was not statistically significant. The final pigment level rose by 17.3%, from an initial measurement of 825.48 µg g^−1^ to 968.08 µg g^−1^, indicating its strong potential to enhance chlorophyll accumulation. Turf also proved effective, with a 25.5% enhancement, reaching 987.41 µg g^−1^ from an initial value of 786.72 µg g^−1^. Comparatively, coco coir + perlite showed a smaller but notable 18.3% increase, from 819.56 µg g^−1^ to 969.37 µg g^−1^, while coco coir alone increased by 9.4%, ending at 751.35 µg g^−1^. In contrast, mixtures such as bark + vermiculite and moss + perlite experienced significant declines. The latter combination showed a 32.8% reduction in chlorophyll level, dropping from 554.20 µg g^−1^ to 372.67 µg g^−1^, while moss + perlite declined by 42.3%, ending at 532.22 µg g^−1^.

In the second trial, (Figure 8B), the total chlorophyll accumulation trends were more pronounced. Pure perlite demonstrated the highest improvement, increasing this pigment content by 90% from 561.04 µg g^−1^ to 1068.05 µg g^−1^, nearly doubling its initial level. Similarly, coco coir + vermiculite showed a significant improvement of 40.5%, reaching a final chlorophyll value of 1061.08 µg g^−1^. Other effective substrates included coco coir + perlite and vermiculite, which increased by 74.6% and 48.9%, reaching final values of 1046.69 µg g^−1^ and 1013.62 µg g^−1^, respectively. Moss + turf also played well, with a notable 66.5% enhancement, ending at 977.70 µg g^−1^. Turf and coco coir demonstrated moderate improvements of 72.3% and 52.9%, with final chlorophyll levels of 909.70 µg g^−1^ and 901.76 µg g^−1^. In contrast, some substrates exhibited limited increases or reductions. For instance, coco coir + moss experienced a decline of 13.4%, dropping from 665.42 µg g^−1^ to 747.51 µg g^−1^, while moss + vermiculite decreased by 18.3%, ending at 778.91 µg g^−1^. Mixtures such as perlite + turf and perlite + vermiculite showed only slight improvements, increasing by 15.2% and 11.4%, with final contents of 723.59 µg g^−1^ and 720.48 µg g^−1^. Overall, perlite, coco coir + vermiculite, and coco coir + perlite proved to be the most effective substrates for enhancing chlorophyll content during this trial.

### 2.8. Carotenoid Content

In the first study, significant variations were observed among substrates, revealing diverse effects on pigment retention and accumulation. Moss exhibited the highest enhancement in carotenoid level, rising by 15.7%**,** from 18.79 µg g^−1^ to 21.74 µg g^−1^**,** followed closely with the use of moss + turf that increased by 6.4%, reaching a final value of 21.83 µg g^−1^. Turf alone also maintained a stable performance, with a slight increase of 2.3%, ending at 21.02 µg g^−1^. These results highlight the effectiveness of moss-containing substrates in promoting carotenoid retention during the early acclimatization stage.

**Figure 9:**
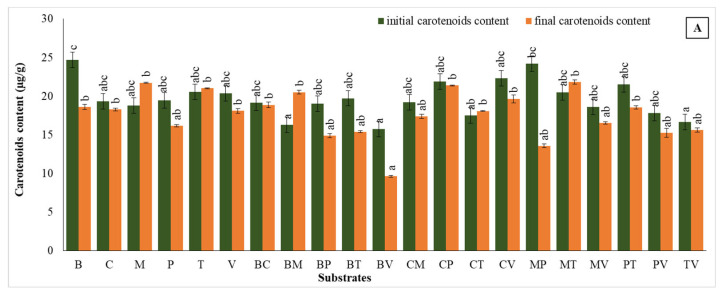

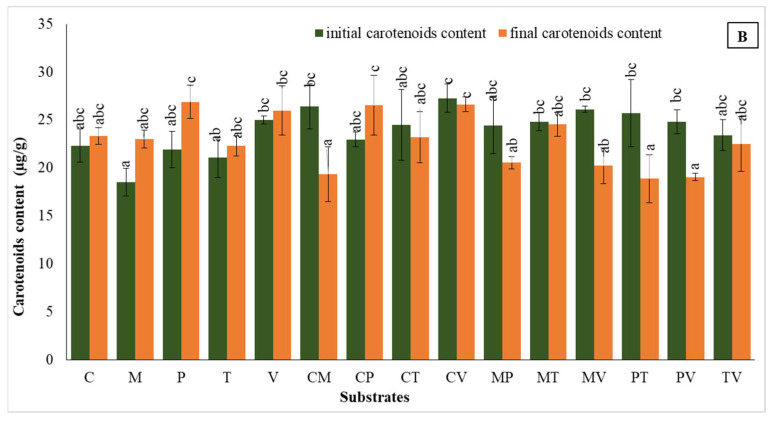
Carotenoids content in the first (**A**) and second (**B**) acclimatization trials, with the use of different substrates. Distinct small vertical letters denote significant differences across treatments (Games–Howell, *p* < 0.05).

In contrast, some substrates experienced notable reductions. For instance, moss + perlite showed the most significant decline, decreasing by 43.9%, from 24.20 µg g^−1^ to 13.58 µg g^−1^. However, bark + moss showed an increase of approximately 26%, and all bark-based mixtures exhibited a carotenoid content reduction at the final measurement with varying percentages. The most pronounced decline was observed in the group of bark + vermiculite (38.6%). Similarly, pure perlite and bark + turf resulted declines of 16.9% and 21.9%, respectively, with final pigment levels of 16.20 µg g^−1^ and 15.41 µg g^−1^. Coco coir mixtures that contained perlite or vermiculite showed relatively stable results, with minimal decreases of 2.2% and 11.8%, ending at 21.41 µg g^−1^ and 19.67 µg g^−1^**.** Coco coir + moss demonstrated a moderate decline of 9.4%**,** while coco coir alone maintained a steady performance with a minor decrease of 5.5%**,** achieving a final carotenoid content of 18.28 µg g^−1^ (Figure 9A).

Overall, moss, moss + turf, and turf were the most effective substrates for promoting carotenoid retention during this acclimatization trial, while combinations involving perlite and bark resulted limited capacity for sustaining carotenoid levels. These findings underscore the critical role of substrate selection in optimizing pigment stability during early plant development.

In the second trial, Perlite exhibited the most significant increase in carotenoid content, rising by approximately 22.8% from an initial value of 21.89 µg g^−1^ to 26.89 µg g^−1^. Similarly, vermiculite displayed a smaller, but consistent, increase of around 3.9%, ending with a final value of 25.97 µg g^−1^. Other pure substrates such as coco coir and turf also showed slight increases in carotenoid content, with increases of 4.4% and 5.6%, respectively. Meanwhile, the coco coir + perlite mixture demonstrated a 15.5% increase, highlighting the effectiveness of this combination under controlled laboratory conditions.

In contrast, several substrates experienced reductions in carotenoid levels. For instance, coco coir + moss decreased sharply by approximately 26.7%, declining from 26.40 µg g^−1^ to 19.35 µg g^−1^. Similarly, perlite mixtures with turf or vermiculite showed reductions of 26.6% and 23.2%, respectively. These results suggest that while some substrate combinations support carotenoid retention or increase, others may limit carotenoid stability, potentially due to nutrient availability or environmental interactions. Moss in combination with vermiculite or perlite also exhibited declines of 22.6% and 15.9%, indicating variability in the performance of moss-based mixtures (Figure 9B).

### 2.9. Survival Rate in the First and Second Acclimatization

A one-way analysis of variance (ANOVA) was conducted to evaluate the impact of different treatments on survival rate during the first and second acclimatization experiments. In the first experiment, the results revealed a highly significant effect of treatments on survival rate (F(20,42) = 118.348, *p* < 0.001). Similarly, in the second experiment, substrate type demonstrated a significant influence on survival rate (F(13,31) = 16.325, *p* < 0.001). Following this, post hoc tests (e.g., Tukey’s HSD) were conducted, which can pinpoint specific differences between substrate groups.

**Figure 10:**
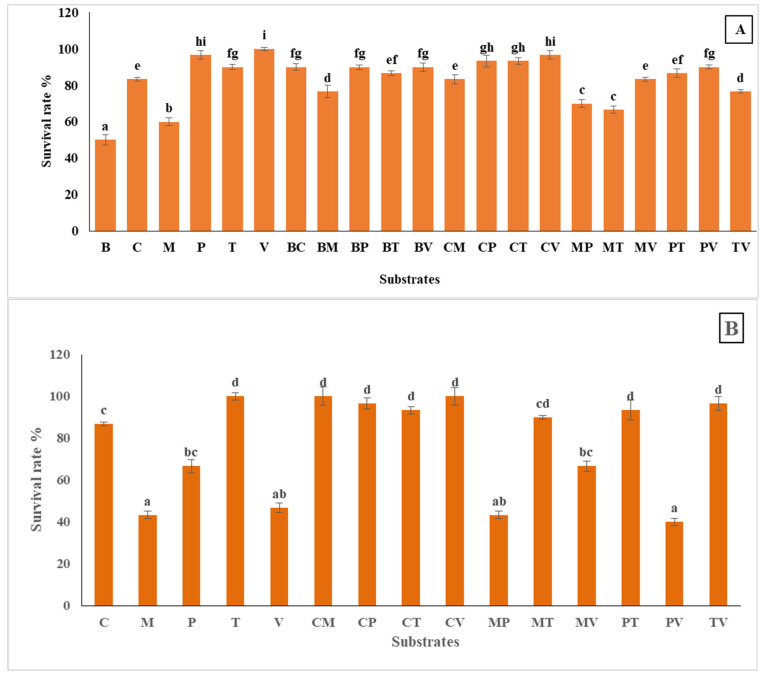
Survival rates in the first (**A**) and the second (**B**) acclimatization trials, with the use of different substrates. Distinct small letters denote significant differences across treatments (Games–Howell, *p* < 0.05).

From the provided figure (Figure 10A,B), the survival rates of plants during the first acclimatization experiment varied considerably across different substrate types, ranging from 50% to 100%. The highest survival rate, of 100%, was observed in the groups of vermiculite, indicating optimal conditions for plant acclimatization. Perlite and coco coir + vermiculite also performed well, with survival rates of 96.67%, while coco coir mixtures with perlite or turf achieved 93.33%. In contrast, bark recorded the lowest survival rate at 50%, highlighting its limited effectiveness. Moss-based substrates, such as moss and its mixtures, contained turf or perlite also showed relatively low survival rates, ranging from 60% to 70% (Figure 10A). Overall, vermiculite, perlite, and specific substrate combinations involving coco coir were among the most effective in enhancing plant survival.

In comparison, during the second trial, similar trends were observed (Figure 10B). Among the pure substrates, turf and coco coir recorded the highest survival rates at 100% and 86.67%, respectively, with turf demonstrating a 1.15-fold increase compared to coco coir. Conversely, vermiculite showed the lowest rate at 46.67%, nearly 0.47-fold of the survival in turf. In mixed coco coir substrates, combinations with perlite or turf achieved maximum survival rates of 100%, significantly outperforming other mixtures like perlite + vermiculite, which had the lowest rate at 40%, a 2.5-fold decrease compared to the top-performing mixes. Notably, coco coir + vermiculite and perlite + turf also demonstrated high survival rates at 93.33%, maintaining a production of over 90% efficiency, while moss + perlite exhibited a reduced rate (43.33%). These results indicate that turf and coco coir, whether used individually or in combination with other substrates, consistently enhance survival rates, suggesting their superior suitability in this context.

## 3. Discussion

Based on the characteristics of the substrates used under both acclimatization conditions in the greenhouse and laboratory (Table 1, Section 4), the results of the experiments emphasize the significant role of substrate composition in influencing shoot development in plants. In the first type of acclimatization performed in a greenhouse (one-step), conducted in a greenhouse setting, the combination of moss and perlite emerged as the most effective substrate, resulting in a 43% increase in shoot numbers. According to [31], this enhanced growth can be attributed to the properties of moss-based mixtures, which provide optimal nutrient availability and facilitate proper gaseous exchange around the roots [32]. Conversely, substrates such as moss and perlite, used separately, and combinations like bark and turf showed little to no growth, or even a decline in shoot numbers. This underperformance may stem from inadequate moisture retention or insufficient nutrient availability, both of which are critical for successful plant establishment.

In two-step acclimatization (initially in the laboratory, followed by greenhouse transfer), the results highlighted the superior performance of turf-based mixtures and coco coir substrates, particularly under controlled conditions, in promoting shoot development. The effectiveness of turf substrates can be linked to their high organic content and the presence of beneficial microbial activity, which enhance nutrient uptake and root establishment [39,40]. Similarly, the performance of coco coir is attributed to its excellent water retention capacity and its ability to support robust root systems, making it a preferred choice for various horticultural applications [32].

During the first acclimatization type, the most significant increases in plant height were observed with the substrates bark + perlite and bark + vermiculite, achieving height increases of 278% and 224%, respectively. Specifically, coco coir alone also demonstrated impressive results in promoting plant height. According to Colombo [41], pine bark enhances the structural properties of the substrate, particularly by increasing porosity, which facilitates effective gaseous exchange between the substrate and the surrounding environment. Martinez et al. [42] further suggest that bark can stimulate plant elongation due to its low moisture content, prompting plants to elongate as a mechanism to access atmospheric humidity through foliar absorption. For instance, *Tillandsia viridiflora* showed an additional 2 cm of growth on a bark-based substrate compared to a perlite and moss mixture.

Under laboratory conditions of the second acclimatization trial, coco coir-based mixtures continued to exhibit superior performance, with the combination of coco coir + vermiculite leading to a 216% increase in plant height. The consistent effectiveness of turf-based and coco coir substrates under controlled conditions highlights their dual benefits: promoting shoot development in the short term, while fostering robust root systems that contribute to long-term plant health [43].

Across both acclimatization types, coco coir and its mixtures, particularly with moss or vermiculite consistently yielded the highest plant weight gains, highlighting their superior support for V. splendens acclimatization. This enhanced performance can be attributed to their ability to provide essential nutrients and maintain adequate moisture levels, which promote leaf expansion and overall plant weight increase [44]. In contrast, turf and perlite mixtures exhibited limited growth, with turf showing a 24.5% decline in plant weight. This observation aligns with Gül et al. [45], who noted that perlite can reduce mass aggregation. Despite these limitations, turf substrates possess beneficial properties, such as a microporous structure and the ability to rehydrate easily after drying [46,47]. These characteristics can create an optimal medium that supports a 100% survival rate during acclimation, minimizing plant stress and thus reducing the need for compensatory growth responses like leaf expansion or water storage [48].

Notably, in the second trial, the coco coir + moss mixture demonstrated the most significant increase in plant fresh weight, with a remarkable 3.76-fold growth. This synergistic effect likely results from enhanced nutrient availability and superior moisture retention, both of which are critical for optimal plant development and successful acclimatization [43].

According to the leaf length during the first acclimatization study, coco coir (both alone and in mixtures) consistently promoted the highest leaf growth rates. Beyond its well-documented role in providing essential nutrients and moisture [44], coco coir also enhances nutrient and oxygen uptake and supports tissue formation [44,46,47], contributing to both plant height and leaf size. Additionally, coco coir improves water uptake and supplies key nutrients, such as potassium and phosphorus, which are vital for leaf tissue development [49]. The combination of coco coir and turf achieved the longest final leaf length, measuring 74.46 mm. Turf, known for maintaining moisture levels and promoting oxygenation [46,47], likely complements coco coir’s properties, resulting in superior leaf growth. In contrast, plants grown in bark substrates produced shorter leaves (37.67 mm). This outcome, while differing from expectations based on plant height [42], may be explained by the structural characteristics of bromeliads, which naturally keep leaves erect, making longer leaves unnecessary for taller growth.

In the second trial, perlite, vermiculite and their mixtures did not favor the leaf length growth. Gül et al. [45] suggest that, under warm conditions, perlite’s limited nutrient retention and reduced mass aggregation negatively affect leaf size. This substrate’s tendency to evaporate water quickly may also force plants to reduce leaf size as a strategy to minimize water loss through evapotranspiration [49]. However, in certain conditions, such as in tomato cultivation, perlite remains an effective soilless substrate [50]. Conversely, coco coir and its mixtures consistently produced better results for leaf length compared to vermiculite alone. According to Caldeira et al. [51], vermiculite requires a balance of essential nutrients and should ideally be used with organic materials to enhance aeration and porosity. This aligns with our findings, where the addition of coco coir to vermiculite significantly improved leaf length in both acclimatization phases, reinforcing the importance of substrate composition in optimizing growth outcomes.

During the first acclimatization, plants grown in moss developed a greater number of roots, with a minimal reduction of 25.5%, although the roots were relatively short. The abundant water availability under greenhouse conditions likely enabled moss to leverage its water retention capacity for growth [46], creating competition between the moss substrate and the bromeliad roots for water. Additionally, the loose structure of moss may have hindered effective attachment, prompting the plants to develop more, smaller roots to enhance anchorage [52].

In the second acclimatization process, the perlite + turf mixture resulted the most roots, showing a significant increase of approximately 35%. This aligns with recommendations from Nawandish et al. [53], who advocate for the use of perlite beds in ex vitro rooting of micro-cuttings due to their effectiveness in promoting high acclimatization rates and healthy root and shoot development in *Pyrodwarf* plantlets. On the other hand, coco coir and its combination with perlite did not significantly increase root numbers. This may be attributed to coco coir’s ability to provide sufficient nutrition and hydration, reducing the need for extensive root proliferation. Similarly, the moss + turf mixture supported steady root development, suggesting that these substrates provide a balanced environment that promotes gradual root establishment. These findings highlight their suitability for the critical transition phase before plants are transferred to greenhouse conditions, ensuring stable growth and effective acclimatization.

In the first trial, the longest average root length was observed in plants that were grown in coco coir + turf mixture, reaching 41.71 mm, followed by coco coir alone and its mixtures with either perlite or vermiculite. A similar trend was noted during the second study, where coco coir and its mixtures consistently produced the longest roots. The superior performance of coco coir in promoting root elongation can be attributed to its ability to provide nutrients through nitrogen immobilization and its strong carbon-to-nitrogen (C:N) ratio, facilitated by the microbiota present [54]. The addition of perlite to coco coir further enhances root growth by increasing substrate porosity and improving root aeration [32,55]. Vermiculite, as a solid substrate, may also contribute to longer root development due to its ability to adhere to roots, providing structural support [56]. This is consistent with the anatomical and morphological characteristics of *Vriesea* species, where roots primarily function as anchorage structures rather than for absorption [57]. In contrast, the shortest average root length (8.07 mm) was observed in bark substrates, possibly due to the substrate’s hydrophobic nature and large particle size, which limited root penetration and nutrient absorption [58]. Pine bark’s hardness may have further impeded root development, thereby affecting the plant’s ability to uptake water and nutrients [59].

Regarding the total chlorophyll content, our findings underscore that, while some substrates effectively support chlorophyll development, others may hinder its retention across both acclimatization types. This pigment level serves as a key physiological marker of plant metabolism, chloroplast functionality, and photosynthetic efficiency. The coco coir + perlite mixture consistently supported high chlorophyll accumulation in both acclimatization trials. This synergy is attributed to coco coir’s nutrient content and perlite’s porosity, which together foster optimal leaf health and darker pigmentation, as noted in studies on strawberries [59,60], *Lilium* [60], and crops such as sunflower and corn [61]. Conversely, the lowest chlorophyll content was observed in plants grown on bark + vermiculite during the first acclimatization, representing a 32.8% reduction. While, in the second trial, perlite + vermiculite showed only a slight increase of 11.4%. These findings suggest that pure vermiculite may contribute to lower chlorophyll accumulation. However, vermiculite’s role as a medium for nutrient transport, particularly in soilless systems, has been associated with increased photosynthetic efficiency due to high enzymatic activity and nutrient conductivity [62,63]. This substrate also enhances potting mixtures by facilitating the transmission of essential nutrients, like iron, potassium, and calcium [64].

Carotenoid accumulation varied between acclimatization phases. In the first study, turf alone and in combination with moss supported robust carotenoid production. During the second acclimatization trial, coco coir mixtures generally showed relatively high carotenoid content, except when mixed with moss. This reduced carotenoid accumulation in the laboratory setting aligns with expectations, as lower but consistent light intensity typically limits carotenoid synthesis [65]. Temperature also plays a crucial role, with lower temperatures promoting carotenoid formation as part of an acclimatization response [22]. However, given the stable conditions in this study, carotenoid levels remained relatively unchanged from the initial stages to the end. Nutrients such as magnesium and nitrogen are known to enhance carotenoid synthesis [66]. Additionally, carotenoid production can vary significantly within the same genus of bromeliads, depending on the species [67]. Further studies with extended experimental durations or additional repetitions may yield more significant differences in carotenoid content, allowing for a deeper understanding of substrate effects on pigment development.

The survival rate of *Vriesea splendens* ‘Fire’ plants was significantly influenced by the substrates used during both acclimatization trials, reflecting their impact on various morphological and physiological traits. Additionally, the acclimatization technique itself plays a crucial role in plant survival. For example, according to Da Silva et al. [68], the survival of *Dendrobium* plantlets can be significantly enhanced by gradually adapting them to an ex vitro environment. This process involves carefully transitioning the plantlets to external conditions, minimizing the risk of desiccation and wilting during the adaptation period.

As one of the main results of the first acclimatization, vermiculite demonstrated a 100% survival rate despite showing moderate performance in other morphological traits. This substrate’s high water holding capacity likely contributed to improved chlorophyll, root, and leaf production, consistent with findings by Pisa et al. [69]. Surprisingly, vermiculite outperformed coco coir in survival, although coco coir-based mixtures still exhibited high rates of acclimatization [28]. Mixtures of coco coir and perlite (93.33%), coco coir plus turf (93.33%), and coco coir with vermiculite (96.67%) provided ideal conditions for plant growth due to their superior moisture retention, nutrient availability and disease suppression [44,61]. Turf-based mixtures, however, showed variable results. When combined with moss, turf resulted in a survival rate of 66.67%, which was lower than expected given its moisture retention properties. This may be attributed to competition between turf and moss, leading to reduced nutrient availability for the bromeliads. Moss alone also performed poorly (60% survival), likely due to its rapid growth outcompeting bromeliads for resources and reducing anchorage efficiency [70]. The bark substrate resulted the lowest survival (50%), due to bark’s hydrophobic properties, which limit water retention and root development [71]. Consequently, bark was excluded from the second acclimatization phase.

In the second trial, vermiculite exhibited one of the lowest survival rates (46.67%), despite its initial success. The decline may be attributed to prolonged moisture retention under low ventilation in laboratory conditions, which negatively impacted plant development. Light intensity may have also played a role, as reduced light can decrease bromeliad productivity [42,72]. In contrast, coco coir-based mixtures demonstrated exceptional results, with coco coir + vermiculite or moss achieving a 100% survival rate. The superior performance of coco coir mixtures is attributed to their balanced porosity [55], nutrient content, and moisture retention, promoting optimal root and shoot development [43,68]. Research supports coco coir’s ability to foster root elongation and plant mass increase due to its carbon and nitrogen availability [61]. Interestingly, pure turf also achieved a 100% survival rate during the second acclimatization trial, though plants exhibited lower biomass. This suggests that under controlled conditions with stable temperature and light, turf provides an ideal environment for acclimatization, albeit without maximizing growth potential [48].

The insights and recommendations generated from this study include that, in both trials of acclimatization, coco coir emerged as the most suitable substrate, consistently supporting robust plant development. While it did not always achieve the highest values in every parameter, it provided favorable outcomes across key metrics such as plant size, leaf and root growth, root number, and overall survival rates. Its nutrient-rich composition and excellent moisture retention make it a versatile choice [73]. For the one-step trial, vermiculite stands out, achieving the highest survival rates. Its water holding capacity and aeration properties proved beneficial in sustaining plant health. In contrast, for the two-step process, turf demonstrated notable success, ensuring 100% plant survival by the experiment’s conclusion. Despite the epiphytic nature of Vriesea splendens and related bromeliads, the results indicate that using pure bark as a substrate is not recommended. Its physical structure impedes root development, limiting water and nutrient absorption. Similarly, while moss naturally coexists with bromeliads, it negatively affected performance in this study, likely due to its competitive use of moisture.

These findings underscore the importance of substrate selection, not only for immediate growth outcomes, but also for sustaining plant vitality over time. Further research is essential to deepen our understanding of the morphological, physiological, and genetic responses of Vriesea splendens during acclimatization. Such studies will not only enhance cultivation techniques for the ornamental plant industry, but also support ex situ conservation efforts, contributing to the preservation of bromeliads and their natural ecosystems.

## 4. Materials and Methods

### 4.1. Plant Material

The study involved cultivating seeds from the *V. splendens* ‘Fire’ cultivar, characterized by its compact growth with leaves measuring 20 to 25 cm and flower stalks 30 to 35 cm tall [74]. Seeds were sourced from a mother plant and grew in vitro in an MS medium at the Hungarian University of Agriculture and Life Sciences. After initial multiplication and rooting, the plants underwent a pre-treatment phase before being transferred to a greenhouse for acclimatization.

### 4.2. Pre-Treatment

**Figure 11:**
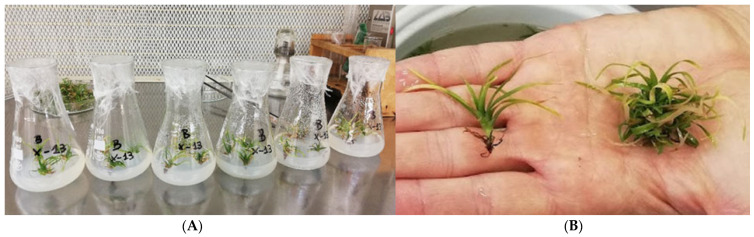
(**A**) Agar medium in flasks containing shoots of *V. splendens* ‘Fire’. (**B**) Close-up view of *V. splendens* ‘Fire’ plant segmentation, displaying the primary plant on the left side and secondary shoot formations on the right side.

As pre-treatment, in vitro shoots were placed in 100 mL Erlenmeyer flasks containing Murashige and Skoog (MS) basal medium [75], fortified with 6.5 g/L agar and 20 g/L sucrose (Figure 11A). The procedure was conducted under sterile conditions in a laminar flow cabinet to ensure contamination-free handling. Each flask held three *V. splendens* ‘Fire’ shoots, ranging in size from 0.5 to 2 cm. Over 700 individual plants were maintained under these controlled conditions for three months at a temperature range of 20–25 °C, with a 16 h daily photoperiod provided by 30-Watt T8 Polylux XL fluorescent lamps, Osram, Munich, Germany (cool and warm white), generating 10 Watts/m^2^, achieving light intensities of 1500–2000 lux.

### 4.3. Selection of Study Groups

During the post-pre-treatment, plants were gently rinsed with tap water to remove residual growth medium from the roots. To prepare specimens for acclimatization, each plant was carefully examined, and multiple shoots were separated using tweezers. Only the primary shoot was retained for each plant to ensure uniformity in growth and survival, though any prominent secondary shoot was preserved to avoid potential risks to the plant’s viability during this separation (Figure 11B).

### 4.4. Acclimatization of *V. splendens* ‘Fire’ in Greenhouse Conditions (in One Step, First Acclimatization)

To begin the acclimatization process, the cleaned and separated plants were transferred to a greenhouse and planted in plug trays containing 104 cells. Each cell was filled with a different substrate or substrate combination to assess plant adaptation. The primary substrates used included turf, vermiculite, perlite, coco coir, pine bark and moss, each offering unique characteristics beneficial to plant growth (Table 1). In total, 630 plants, divided into groups of 30, were tested across 21 substrate treatments.

**Figure 12:**
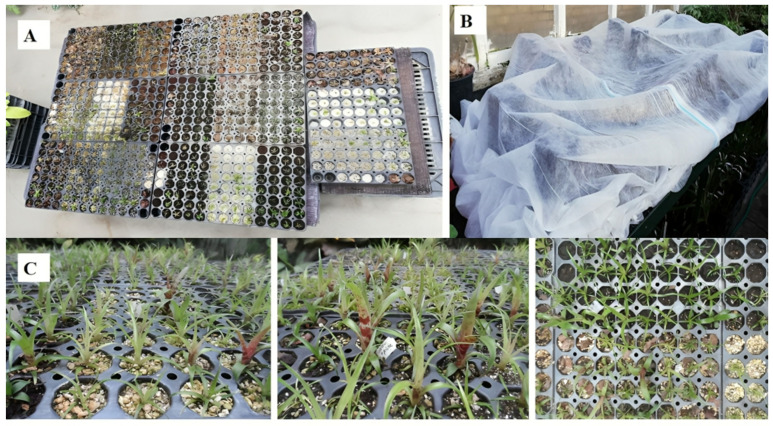
(**A**) Various substrate types prepared in trays prior to planting *V. splendens ‘Fire’* groups. (**B**) A fiber cover was used during the first three weeks to maintain optimal humidity levels for the sensitive plants. (**C**) Close-up view of acclimatized *V. splendens ‘Fire’* plants after a 5-month period.

Substrate combinations tested in plug trays: Table 2 and Figure 12A below details the 21 substrate combinations tested, with each combination tailored to enhance specific growth characteristics in *V. splendens ‘Fire’*. Each substrate and combination was carefully selected to explore optimal growth conditions, balancing factors such as nutrient availability, moisture retention, and aeration, ultimately aiming to improve *V. splendens ‘Fire’* acclimatization success in greenhouse settings.

As following planting, a fiber cover was applied for the initial three weeks to maintain adequate humidity levels (Figure 12B). After this period, all plants were cultivated under standardized greenhouse conditions, receiving uniform irrigation across all substrate treatments. Irrigation was manually managed to maintain soil moisture levels close to the pots’ water holding capacity. Soil moisture and soil water potential (kPa) for each pot were monitored daily using a Blumat Digital PRO Plus instrument (Blumat GmbH and Co. KG, Telfs, Austria).

Growth occurred under natural light, with temperature maintained between 18 and 27 °C, closely matching laboratory pretreatment conditions. At the end of a five-month period, physical and physiological characteristics were assessed, marking the completion of the acclimatization phase (Figure 12C).

### 4.5. Two-Step Acclimatization of V. splendens *‘Fire’* in Laboratory and Greenhouse Conditions

For the second acclimatization phase, a two-step process was implemented. Based on initial acclimatization results, coconut coir, turf, perlite, and vermiculite substrates and their respective combinations were selected. Pine bark and moss were excluded due to their unfavorable effects, such as low plant survival rates (approximately 50%) and limited growth potential. Typically, these substrates are mixed with others like sand or turf to improve humidity and bulk density [37].

#### Acclimatization Process

**Figure 13:**
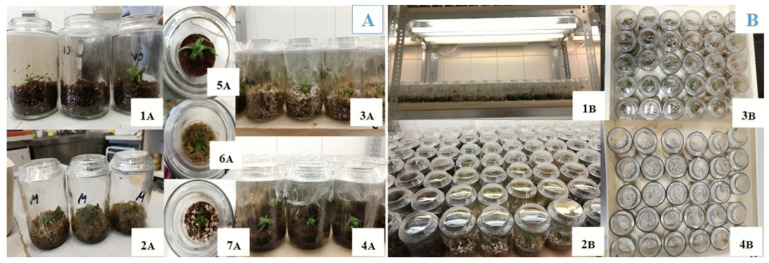
(**A**) Top view of *V. splendens ‘Fire’* individuals cultivated in glass jars under laboratory conditions. 1A: coco coir + vermiculite, 2A: moss, 3A: perlite + moss, 4A: coco coir, 5A: turf, 6A: moss, 7A: perlite + coco coir. (**B**) *V. splendens ‘Fire’* individuals cultivated in glass jars under laboratory conditions. 1B: Glass jars arranged on shelves under controlled lighting. 2B: Close-up of vessels covered with foil wrap. 3B: Planted glass jars with moss + perlite (top view). 4B: Empty glass jars with perlite (top view).

1. Laboratory phase: cleaned and separated shoots were planted in 200 mL glass jars filled with one of the 10 substrate combinations listed below, in Table 3. Each jar was covered with a double layer of foil to maintain humidity (Figure 13(4B)). Plants were grown in a culture room under pre-treatment lighting and temperature conditions (as detailed in Section 2.2) for three months (Figure 13).
2. Greenhouse phase: plants with developed root systems were then transferred to 104-cell plug trays, using the same substrate types from the laboratory phase. After two months, the surviving plants were assessed based on morphological and physiological parameters, consistent with the first acclimatization trial. The entire process spanned five months, including both steps. Each substrate treatment was applied to 30 plants, totaling 300 plants in this trial. Morphological and physiological data were collected at the end of the five-month period, providing comparative insights between the first and second acclimatization trials.

### 4.6. Morphological and Physiological Characteristics of V. splendens Measured Both After Pre-Treatment and at the End of the Acclimatization Process, for Both the First and Second Phases

#### 4.6.1. Morphological Features

The following physical parameters were recorded.

Shoot count: the number of shoots was manually counted. Plant height: measured in mm using a standard ruler, focusing on the longest leaf per shoot in the vertical direction. Leaf length: the largest leaf’s length was also measured. Fresh weight: the weight of the entire plant was determined using an analytical balance, recorded in grams. Root characteristics: the number of roots emerging from the shoot and length of roots were assessed, with root length measured from the base of the leaves to the longest primary root.

#### 4.6.2. Physiological Features

##### Pigment Extraction

For pigment extraction, approximately 100 mg of leaf tissue from each plant group (categorized by substrate type) was used. The leaves were crushed in a ceramic mortar with 0.5 g of quartz sand and 10 mL of 80% acetone. The mixture was refrigerated for 24 h at 4 °C, then absorbance was measured at 644, 663, and 480 nm using a GeneSys VIS-10 spectrophotometer (Thermo Fisher Scientific Inc., Waltham, MA, USA). Leaf pigment concentrations were determined as follows:

Chlorophyll concentration (µg g^−1^): was calculated using the formula:

$$(20.2\times A644+8.02\times A663)\frac{V}{W}$$

where “V” is the volume of the tissue extract (10 mL), “W” is the fresh weight of the tissue (0.1 g), and “A” represents absorbance at 644 nm and 663 nm [76].

Carotenoid concentration (µg g^−1^) was determined using the formula:

$$\frac{5.01\times A480}{W}$$

where “A480” is the absorbance at 480 nm [76].

### 4.7. Survival Rate

The survival rate of plants after the 5-month acclimatization period was calculated as the percentage of plants still alive at the end of both acclimatization trials.

### 4.8. Statistical Analysis

Statistical analyses were conducted using SPSS Statistics 23.0 (IBM Corp., Armonk, NY, USA). One-way multivariate analysis of variance (MANOVA) was conducted to evaluate the effects of different treatments on all measured parameters, except for the survival rate (%), which was analyzed using one-way analysis of variance (ANOVA). The normality of the residuals for the treatments was assessed using the Kolmogorov–Smirnov test, which confirmed normality (*p* > 0.05). Additionally, the homogeneity of variances among treatments was verified using Levene’s F-test, and this assumption was also satisfied (*p* > 0.05). Mean comparisons were performed using Tukey’s HSD test [77] to determine significant differences, based on homogeneity of variances, with a significance level set at 5% (*p* < 0.05). Grouping letters were assigned to indicate statistical differences among treatments within the same stage of development, ensuring comparisons were made only within the same time point.

## 5. Conclusions

The acclimatization of *Vriesea splendens* ’Fire’ demonstrated that substrate choice significantly affects plant growth and survival. In the first trial, moss + perlite enhanced shoot numbers, while bark + perlite increased plant height. Coco coir mixtures, particularly with vermiculite, consistently improved plant weight, leaf length, and root length. Conversely, bark substrates underperformed across most metrics. In the second trial, turf and coco coir mixtures achieved a 100% survival rate, though turf alone resulted in lower plant weight. Physiological traits, including chlorophyll and carotenoid content, showed minimal variation, indicating a need for extended studies to observe more pronounced effects. Coco coir-based mixtures proved to be the most effective overall, supporting robust growth and high survival rates. Future research should explore longer experimental durations to optimize acclimatization strategies.

## Figures and Tables

**Table 1:** Characteristics of primary substrates for *V. splendens* acclimatization.

Substrate	Characteristics	References
Turf	Partially decomposed organic matter from plants, high in organic nutrients and provides a stable structure for plant growth; supports growth with or without microorganisms.	[33,34]
Vermiculite	Mineral substrate rich in iron and magnesium silicates, offering structure and high water retention; facilitates natural mycorrhizal growth, enhancing nutrient supply and plant survival.	[34]
Perlite	Foamy, amorphous fragments from volcanic rock, promoting aeration and porosity, and increasing water retention capacity.	[35]
Coco Coir	Fibers from coconut husks containing potassium, zinc, iron, manganese, and copper; lignin content supports lignocellulose development in plants.	[36]
Pine Bark	Woody outer cortex from Pinus species, acidic and moisture-retentive but with low water retention.	[37]
Moss	Hyaline cells in leaves allow water accumulation, providing humidity for plant growth.	[38]

**Table 2:** Substrate combinations used in greenhouse plug trays.

Substrate Combination		Composition	Substrate Combination		Composition
1	100% turf	Turf only	12	50% vermiculite + 50% perlite	Mixed
2	100% vermiculite	Vermiculite only	13	50% vermiculite + 50% coco coir	Mixed
3	100% perlite	Perlite only	14	50% vermiculite + 50% pine bark	Mixed
4	100% coco coir	Coco coir only	15	50% vermiculite + 50% moss	Mixed
5	100% pine bark	Pine bark only	16	50% coco coir + 50% perlite	Mixed
6	100% moss	Moss only	17	50% coco coir + 50% pine bark	Mixed
7	50% turf + 50% vermiculite	Mixed	18	50% coco coir + 50% moss	Mixed
8	50% turf + 50% perlite	Mixed	19	50% perlite + 50% pine bark	Mixed
9	50% turf + 50% coco coir	Mixed	20	50% perlite + 50% moss	Mixed
10	50% Turf + 50% pine bark	Mixed	21	50% moss + 50% pine bark	Mixed
11	50% turf + 50% moss	Mixed			

**Table 3:** Substrate combinations for two-step acclimatization of *V. splendens* ‘Fire’.

Substrate Combination		Composition
1	100% coco coir	Pure coco coir
2	100% turf	Pure turf
3	100% perlite	Pure perlite
4	100% vermiculite	Pure vermiculite
5	50% coco coir + 50% turf	Mixed
6	50% coco coir + 50% perlite	Mixed
7	50% coco coir + 50% vermiculite	Mixed
8	50% turf + 50% perlite	Mixed
9	50% turf + 50% vermiculite	Mixed
10	50% perlite + 50% vermiculite	Mixed

## Data Availability

The data are contained within the article and are available upon request.
